# Investigation of Vocal Fatigue Using a Dose-Based Vocal Loading Task

**DOI:** 10.3390/app10031192

**Published:** 2020-02-10

**Authors:** Zhengdong Lei, Laura Fasanella, Lisa Martignetti, Nicole Yee-Key Li-Jessen, Luc Mongeau

**Affiliations:** 1 Department of Mechanical Engineering, McGill University, Montreal, QC H3A 0C3, Canada;; 2 School of Communication Sciences and Disorders, McGill University, Montreal, QC H3A 1G1, Canada;

**Keywords:** vocal fatigue, vocal distance dose, neck surface accelerometer

## Abstract

Vocal loading tasks are often used to investigate the relationship between voice use and vocal fatigue in laboratory settings. The present study investigated the concept of a novel quantitative dose-based vocal loading task for vocal fatigue evaluation. Ten female subjects participated in the study. Voice use was monitored and quantified using an online vocal distance dose calculator during six consecutive 30-min long sessions. Voice quality was evaluated subjectively using the CAPE-V and SAVRa before, between, and after each vocal loading task session. Fatigue-indicative symptoms, such as cough, swallowing, and voice clearance, were recorded. Statistical analysis of the results showed that the overall severity, the roughness, and the strain ratings obtained from CAPE-V obeyed similar trends as the three ratings from the SAVRa. These metrics increased over the first two thirds of the sessions to reach a maximum, and then decreased slightly near the session end. Quantitative metrics obtained from surface neck accelerometer signals were found to obey similar trends. The results consistently showed that an initial adjustment of voice quality was followed by vocal saturation, supporting the effectiveness of the proposed loading task.

## 1. Introduction

Vocal fatigue may be diagnosed through a series of voice symptoms, which include, for example, hoarse and breathy vocal qualities, pitch breaks, reduced pitch and loudness ranges, throat discomfort, and unsteady voice [1]. Vocal fatigue may be experienced by any individuals during their life time, but it is more frequently encountered by professional voice users in occupational settings. Vocal fatigue increases vocal effort and decreases speaking stamina. Ultimately, vocal fatigue can lead to voice disorders, such as vocal hyperfunction or vocal nodules. Vocal fatigue is difficult to define because many factors, such as self-reported feelings, doctor-rated symptoms, and instrumental measures, could be criteria for its determination. For example, a self-reported feeling of vocal fatigue might be due to psychological stress, thereby not causing much detectable change in physiological measures [2]. Standards for assessing vocal fatigue are therefore difficult to establish. Nevertheless, most current research adopted the definition of vocal fatigue as a sense of increased vocal effort [3].

Considerable progress has been made in the evaluation of voice quality. Perceptual, acoustic, and aerodynamic measurements, along with self-administered tests, have been used to characterize changes in voice quality and performance in laboratorial settings [4,5]. The most commonly used method of voice quality evaluation in clinics is auditory perception, which relies on listeners’ personal experience and expertise. Commonly used subjective evaluation tools included the GRBAS (Grade, Roughness, Breathiness, Asthenia and Strain) proposed by the Japan Society of Logopedics and Phoniatrics, the CAPE-V (Consensus Auditory-Perceptual Evaluation of Voice) proposed by the American Speech-Language and Hearing Association, and the SAVRa (Self-Administrated Voice Rating) proposed by the National Center for Voice and Speech in the United States. These tools require specific vocal stimuli. For example, the CAPE-V requires the completion of three defined phonation tasks assessed through perceptual rating. This therefore limits the applicability of these tools in situations where the vocal stimuli are varied or unspecified. Many studies have investigated uncertainties in subjective judgment methodologies for voice quality evaluation. Kreiman and Gerratt investigated the source of listener disagreement in voice quality assessment using unidimensional rating scales, and found that no single metric from natural voice recordings allowed the evaluation of voice quality [6]. Kreiman also found that individual standards of voice quality, scale resolution, and voice attribute magnitude also significantly influenced intra-rater agreement [7]. Objective metrics obtained using various acoustic instruments have been investigated, and attempts have been made to correlate these with perceptual voice quality assessments [8–12].

A plethora of temporal, spectral, and cepstral metrics have been proposed to evaluate voice quality [13,14]. Commonly used features or vocal metrics include fundamental frequency (*f*0), loudness, jitter, shimmer, vocal formants, harmonic-to-noise ratio (HNR), spectral tilt (H1–H2, harmonic richness factor), maximum flow declination rate (MFDR), duty ratio, cepstral peak prominence (CPP), Mel-frequency cepstral coefficients (MFCCs), power spectrum ratio, and others [15–19]. Self-reported feelings of decreased vocal functionality have been used as a criterion for vocal fatigue in many previous studies [1,4,20–22]. Standard self-administered questionnaires, such as the SAVRa and the Vocal Fatigue Index (VFI), have been used to identify individuals with vocal fatigue, and to characterize their symptoms [23–25]. Hunter and Titze used the SAVRa to quantify vocal fatigue recovery based on 86 participants’ tracking reports. The results showed a self-reported 50% recovery within 4–6 h, and 90% recovery within 12–18 h [24]. Halpern et al. used one of the three dimensions in SAVRa, i.e., the inability to produce soft voice (IPSV), to track vocal changes in school teachers. The SAVRa scores were then compared with two clinicians’ ratings of the participants’ *f*0, and loudness [26]. The overall correlation between self-ratings and clinician ratings was not significant. The average absolute difference score was 1.7. This showed that the clinicians and the teachers had different rating standards.

Prolonged or inappropriate voice use is commonly regarded as one of the causes of vocal fatigue. Vocal loading tasks (VLTs) are often used to investigate the relationship between voice use and vocal fatigue in laboratorial settings [1]. Previous VLT studies have typically instructed participants to complete standardized reading tasks of prescribed durations at specific loudness levels [1,4,24]. Perceptual, acoustic, and aerodynamic measurements have been used to evaluate changes in voice quality and performance before and after VLTs [4]. Unfortunately, the findings from these studies were often reported as inconsistent. Comparisons across different studies have been at times contradictory. This may have been caused by multiple factors: (1) the prescribed VLT might not have induced a detectable level of vocal fatigue across individuals, (2) the amount of vocal loading across participants may not have been consistent due to the lack of a universal method to quantify vocal loading, and (3) there may have been variability in experimental settings. A more robust vocal loading protocol for VLT is therefore needed to improve consistency, and to allow comparisons across different studies.

Amongst methods of quantifying voice use for vocal fatigue assessment, the vocal distance dose, *D*_*d*_, first proposed by Titze et al., was adopted in the present study [27]. The vocal distance dose attempts to approximately quantify the distance traveled by vocal folds during vocal oscillation [27,28]. It is usually calculated in terms of the fundamental frequency, the sound pressure level, the voicing duration, and the vocal duty ratio. Whether *D*_*d*_ correspondes to the true cumulative vocal fold displacement has not yet been verified. But, as a four-parameter estimates, vocal distance dose is more comprehensive than other metrics that are based on one single parameter. Svec et al. described the procedures to calculate the distance dose using synchronized microphone and EGG data [29]. The EGG signal was used to locate the peak position for each vocal cycle in the time domain. The microphone signal was used to quantify loudness. Carroll et al. used cumulative vocal dose data correlations with subjective measurements in vocal fatigue experiments. An abrupt increase in vocal loading was closely related to a harsher subjective self-reported rating [30]. Echternach et al. found that a 10-min intensive VLT with a > 80 dB loudness level was comparable to a 45-min teaching task in terms of vocal dose [31]. Remacle et al. showed that kindergarden teachers had significantly greater distance doses than elementary school teachers based on an investigation of 12 kinderdarten and 20 elementary school female teachers [5]. Bottalico and Astolfi calculated the vocal distance dose and the sound pressure level (SPL) for school teachers during their daily teaching assignments. They found that female teachers had on average a higher (> 3.4 dB) loudness level than male teachers, but vocal distance doses did not differ very much between female and males teachers [32]. Morrow and Connor used an ambulatory phonation monitor to record and calculate the SPL and the distance dose for elementary music teachers with and without voice amplification [33]. The results showed that voice amplification significantly decreased the average SPL and the distance dose. These studies used the vocal distance dose as a quantitative measure of voice use in the VLTs or routine phonation tasks. Despite progress, no definitive correlations have been yet made between subjective assessments and objective measures in vocal fatigue studies. The distance dose prospectively offers a quantitative metric for vocal loading. Such framework is essential for cross-participants or cross-sessions comparisons to be meaningful and reasonable.

In the present study, a uniquely designed VLT was investigated. Ten human subjects were recruited and participated in the study. The vocal distance dose was used to quantify the participants’ vocal loading online during the experiment. Subjective and objective measures were used to assess participants’ voice qualities. A cross-session comparison was made to investigate the relationship between total distance dose and voice quality.

## 2. Research Hypothesis and Objective

We hypothesized that vocal fatigue during dose-based VLT varies with the vocal distance dose, *D*_*d*_. The objective of this study was to investigate possible correlations between auditory-perceptual ratings, self-reported ratings, and acoustic measures during a dose-monitored VLT.

## 3. Participant Recruitment

The human research ethics protocol (A09-M46–11A) was approved by the Institutional Review Board at McGill University. No occupational voice users such as singers, teachers, and voice actors were recruited, because previous studies showed that their voice had greater endurance for vocal loadings than normal voice users [1,34]. The purpose of the study was not communicated to the participants before the experiment was concluded. The participants were only informed that they had to perform reading sessions. This was to help reduce the participants’ biases towards the SAVRa ratings. For example, if the participants were aware that the study was measuring vocal fatigue, they would expect their voice quality to degrade throughout the sessions. This may increase the risk of biased ratings through the introduction of a psychological variable into the cross-session analysis of the participants’ voice quality.

Participants were recruited in Montreal, Canada. The inclusion criteria were that the participants should be female, native English speakers with no history of voice disorders. Sex and gender differences in voice performance were found to be significant by Hunter et al. [35,36]. The present study only used female participants to exclude sex as a variable in the pilot study. Male participants will be used in future experiments. The participants’ demographic information is shown in Table 1. The experiments were conducted in a sound-proof voice recording studio located in the Centre for Interdisciplinary Research in Music Media and Technology (CIRMMT). All experiments took place in the morning, around 9:00 am. The participants were instructed not to use their voice often over a period of 8 h before the experiments. The participants were required to withdraw from the study if they were found to have a voice problem, such as cough and cold, in the early morning. During the experiment, participants who reported any severe physical discomforts, such as unceasing cough and voice loss, were asked to withdraw from the recording session.

## 4. Experimental Protocol Design and Data Acquisition

### 4.1. Vocal Loading Protocol

**Figure 1.**
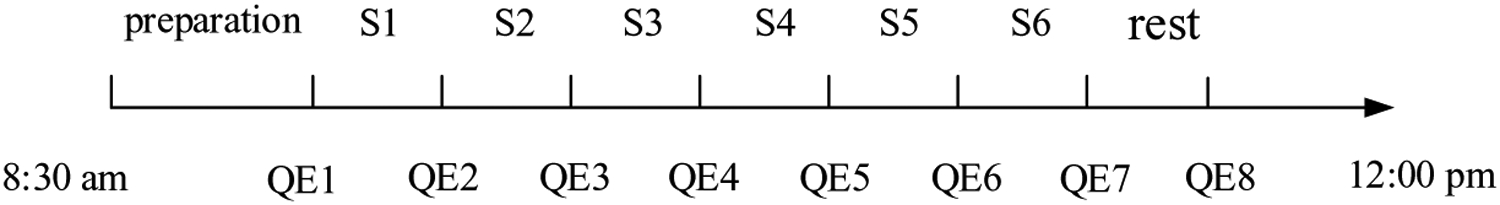
Measurement protocol of the vocal loading task in the vocal fatigue study. QE_i_(i = 1, 2, .., 8): Quality Evaluation; S_i_(i = 1, 2, .., 6) represents each vocal loading task session.

The VLT protocol is illustrated in Figure 1. Before the formal recording, the participants were required to attend a preparation session, during which they learned how to use a voice biofeedback monitor for the experiment. The VLT was structured as a series of six successive sessions (S_i_(i = 1,2,..,6)). The participants were asked to read loudly the novel “Harry Potter and the Sorcerer’s Stone” [37]. For each session, the participants were required to reach a *D*_*d*_ of 500 meters within 25 min. After this reading task, the participants were required to finish a voice quality evaluation test with in 5 min. In preliminary study, a *D*_*d*_ of 500 meters in 25 minutes was found to be intensive enough to induce vocal fatigue on participants, as self-reported. All the participants’ voice was fully recovered after one day. This indicated that the selected *D*_*d*_ level did not induce any long-term vocal damage to the participants. The preliminary study also showed that the distance dose was sensitive to *f*0, loudness and phonatory style. A reading task using a habitual *f*0 and SPL yielded a distance dose of approximately 5 meters per 20 seconds. But a note sung with similar *f*0 and SPL yielded a distance dose over 20 meters per 20 seconds. Therefore, singing was prohibited in this experiment. The participants were asked to use a daily speech communication style.

### 4.2. Voice Biofeedback Monitoring

**Figure 2.**
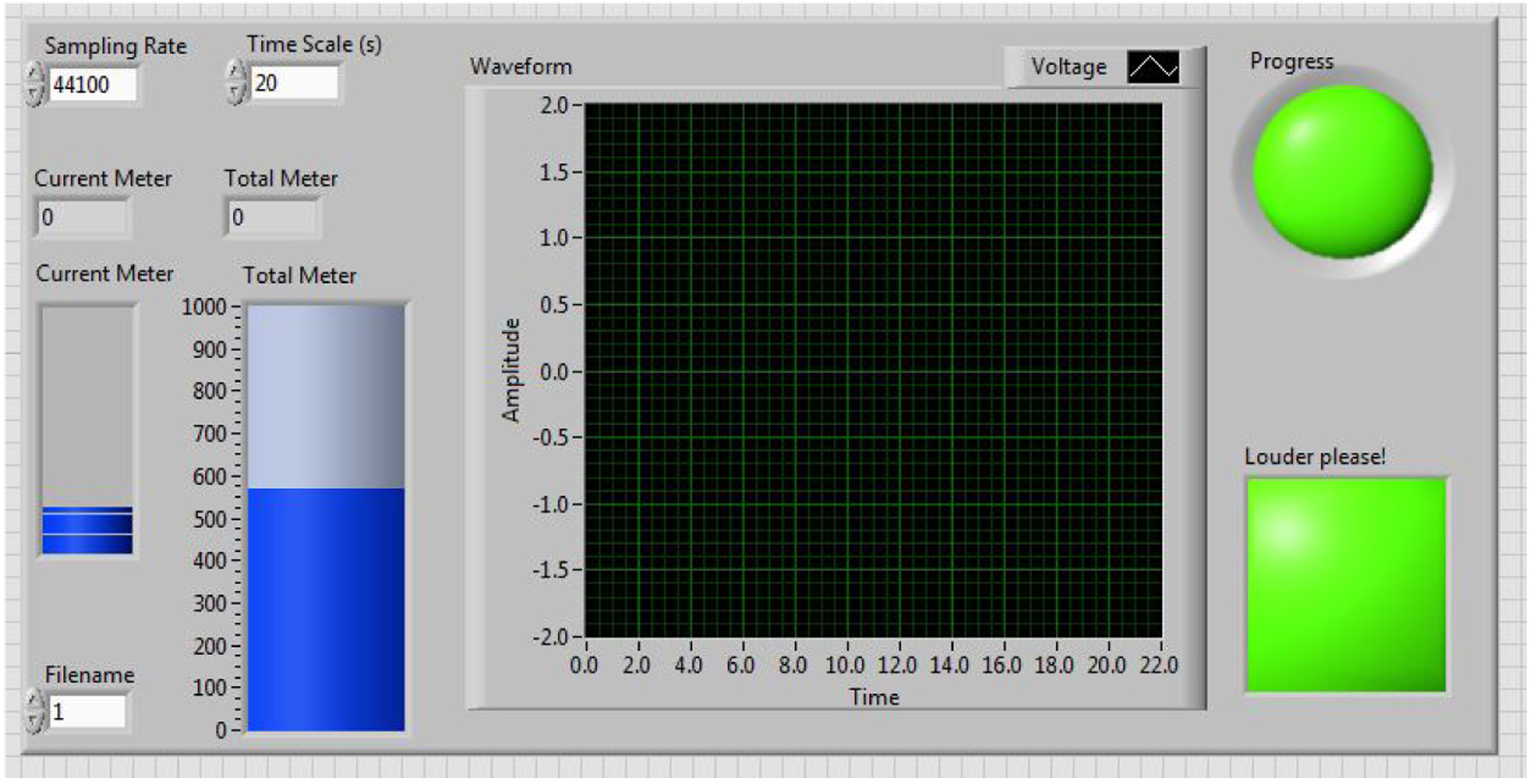
Virtual vocal distance dose monitor.

**Figure 3.**
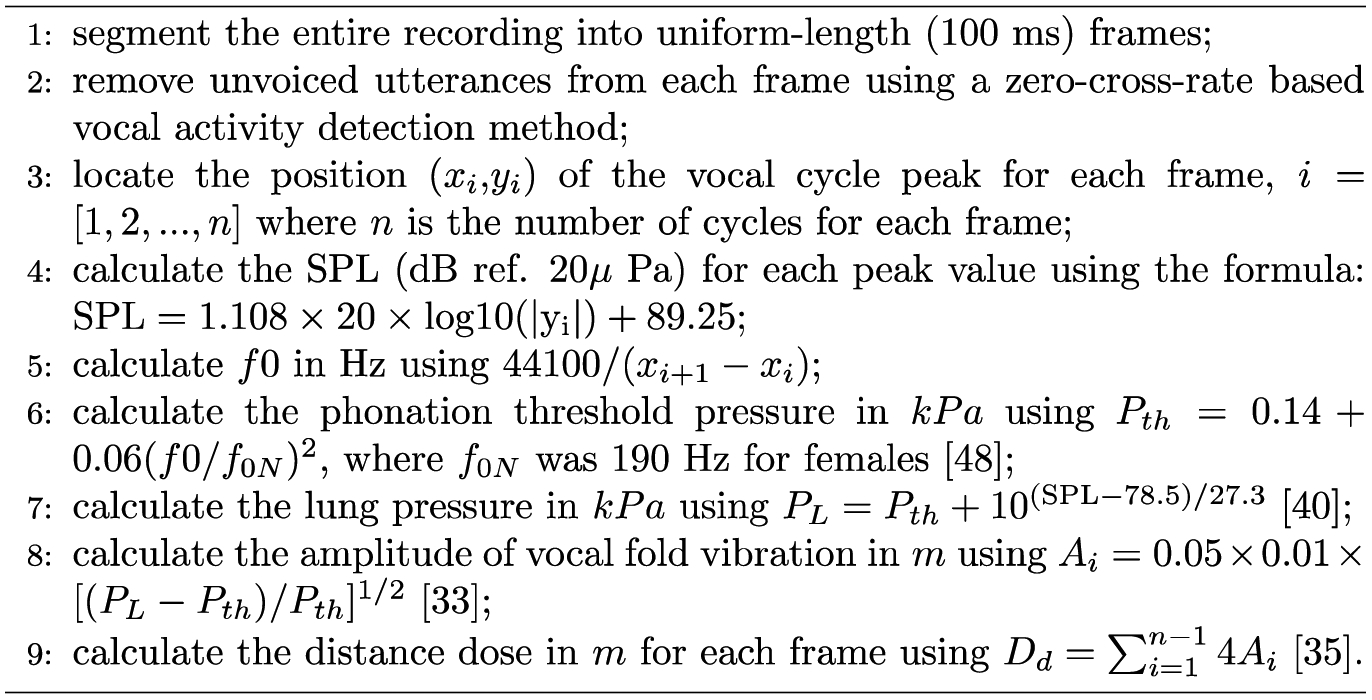
Procedure for the 20 s distance dose calculation

The participants were seated in front of a microphone, and wore a neck surface accelerometer (NSA) mounted using adhesive (*Tensive*) [38]. The NSA recorded the neck surface vibration and streamed it on a hard disk [39–41]. During recording, the participants were asked to remain stationary, with the microphone at a distance of 50 cm from their mouth. The VLT sessions were monitored using a short-time (20 s long) distance dose calculator and a accumulative distance dose calculator in *LabView* (2018, NI, TX, US). A screenshot of the tool is shown in Figure 2. When the cumulative distance dose reached 500 m, the circular progress LED indicator turned from green to red, indicating the completion of one VLT session. The square-shaped LED indicator turned from green to red if the participant’s previous 20 s distance dose did not reach a threshold value of 11 m. The threshold value was adjusted by trial-and-error to be high enough to induce vocal fatigue while ensuring that all participants could complete the VLT sessions. The participants were asked to keep an eye on the two indicators during the VLT sessions, so that they could adjust their vocal effort online, in real time. The virtual vocal distance dose monitoring tool prompted participants to read intensively throughout the VLT sessions. The 20 s distance dose calculation algorithm is illustrated in Figure 3. The total distance dose was calculated as the sum of all previous 20 s distance doses. The recording devices were a condenser acoustic microphone (Type 4178, *Brüel* & *Kjær*, Denmark) and the NSA. The microphone sensitivity was verified using a calibrated precision sound pressure level meter (Type 2250-L, *Brüel* & *Kjær*, Denmark).

### 4.3. Subjective and Objective Measures of Vocal Fatigue

Three subjective methods of voice quality assessment were used in the experiment. They were the fatigue-indicative symptom documentation, the CAPE-V rating, and the SAVRa. Fatigue-indicative symptoms, such as cough, swallowing, and voice break, were recorded manually. Before, between, and after each VLT session, the participants performed a voice quality evaluation (QE) task, rated using the CAPE-V rating [42] and the SAVRa rating [23]. The CAPE-V ratings included six dimensions: overall severity, roughness, breathiness, strain, pitch, and loudness. The SAVRa ratings included three dimensions: speaking effort level (EFFT), laryngeal discomfort level (DISC), and inability to produce soft voice (IPSV). The QE took less than 5 minutes to complete. After completion, the participants remained silent until the start of the next session. A fixed volume (100 ml) of water was given to the participants immediately before each QE. No drinking was allowed during the VLT sessions. After the experiment, the microphone recordings of the CAPE-V task were sent to four certified speech language pathologists (SLPs) for auditory-perceptual rating. The four SLPs had more than 3 years of experience on voice research and clinical diagnosis, and they used the CAPE-V on voice patients and experimental participants quite often during the past three years. Three of them were voice doctors in the McGill University Health Centre, and the other one was a voice research associate in McGill University. The CAPE-V recordings were blindly rated by the SLPs. There were eight audio recordings for each participant. The order of within-participant files was randomized to reduce the SLPs’ rating bias. The audio files were rated participant by participant, so that the SLPs could identify the participant-specific baseline of voice quality for each rating task.

Voice features, such as *f*0, SPL, duty ratio, CPP, spectral tilt, HRF, jitter, and shimmer extracted from the recorded NSA and microphone signals, were labeled with the corresponding session numbers. These voice features were compared across sessions to track the variations between the participants’ voice qualities. These vocal metrics were also compared with the CAPE-V and SAVRa ratings.

### 4.4. Data Analysis

The mean values and standard deviations of the normalized SAVRa and the original CAPE-V rating scores were calculated. The SAVRa scores were first normalized for each participant, and then the normalized scores were clustered for statistical analysis. The scores in the SAVRa and CAPE-V ratings are inversely related to the voice quality, i.e., a higher score implies lower voice quality and vice-versa. This rule applies to all dimension ratings of the SAVRa and CAPE-V. For correlation analysis, all voice quality data were assumed to obey a Gaussian distribution. A rigorous validation of this assumption would require a larger data set, which was beyond the scope of the present study. A Pearson correlation analysis was done between each pairs of dimensions of the SAVRa rating scores and the CAPE-V rating scores.

Fifteen acoustic features were calculated from the microphone and the NSA data using Matlab (2018a). The symbols and a description of the features are shown in Table 2. Features commonly used in previous vocal fatigue studies were selected. The original microphone and NSA signals were segmented into uniform frames of duration 100 ms (20–40 vocal cycles). Frames that were too short (20–30 ms) were insufficient for extracting effective jitter and shimmer, and frames that were too long (> 500 ms) could not satisfactorily resolve *f*0 variations. The unvoiced frames were detected and removed using the zero-cross rate method [43].

The procedure of multivariate analysis in the present study was that a trend was searched in SAVRa scores at first to build a baseline of identifying vocal fatigue. A similar trend in the CAPE-V scores was then searched to verify the consistency between self-administered and SLP-rated methods. Finally, this trend was searched in the fifteen acoustic features to demonstrate some specific features have the potential of indicating vocal fatigue.

## 5. Data Analysis Results and Discussions

### 5.1. SAVRa and CAPE-V Rating Results

**Figure 4.**
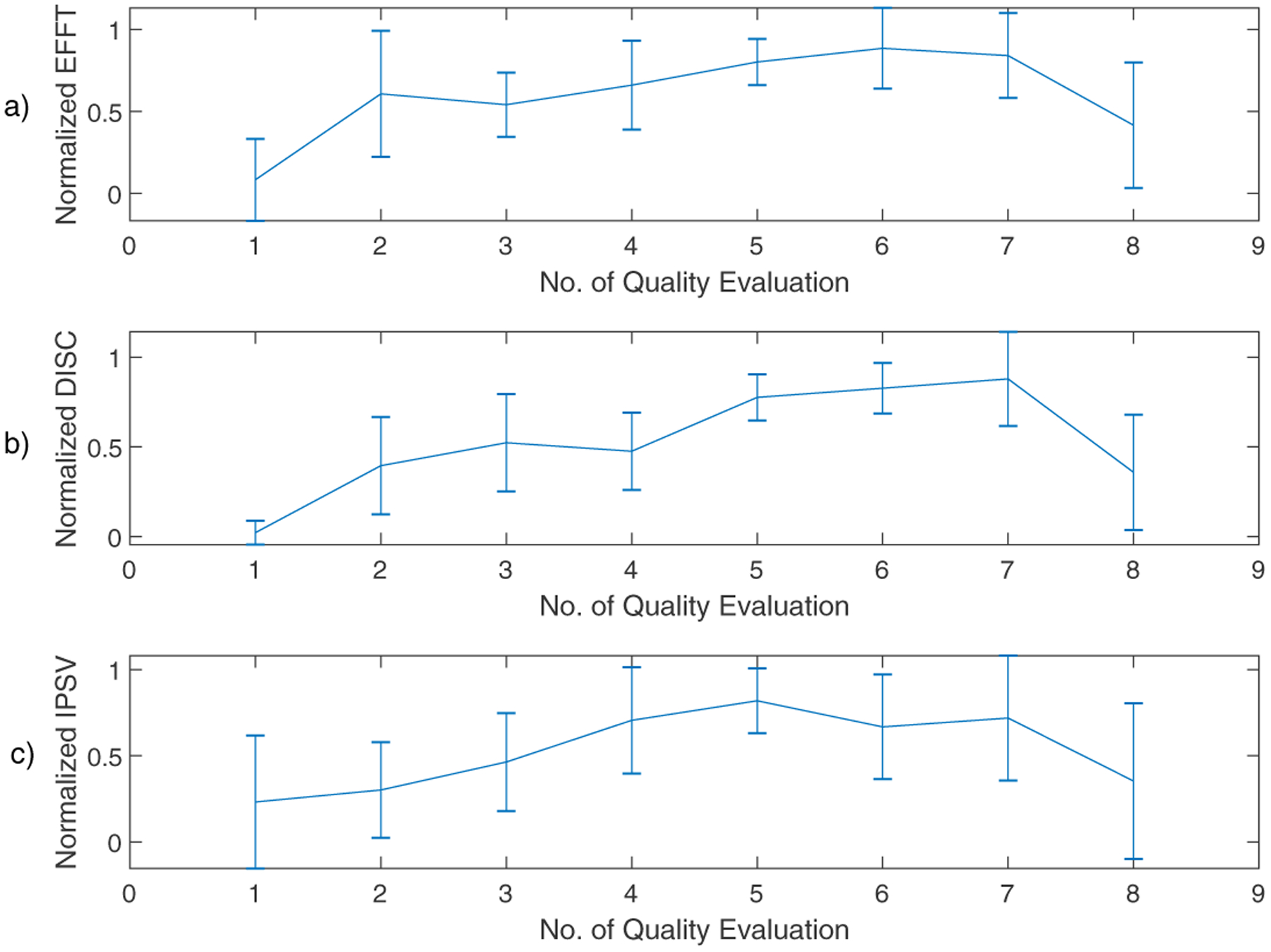
Cross-session variations mean and standard deviation values of SAVRa ratings for all participants (n = 10). (**a**) speaking effort level (EFFT); (**b**) laryngeal discomfort level (DISC); (**c**) inability to produce soft voice (IPSV).

**Figure 5.**
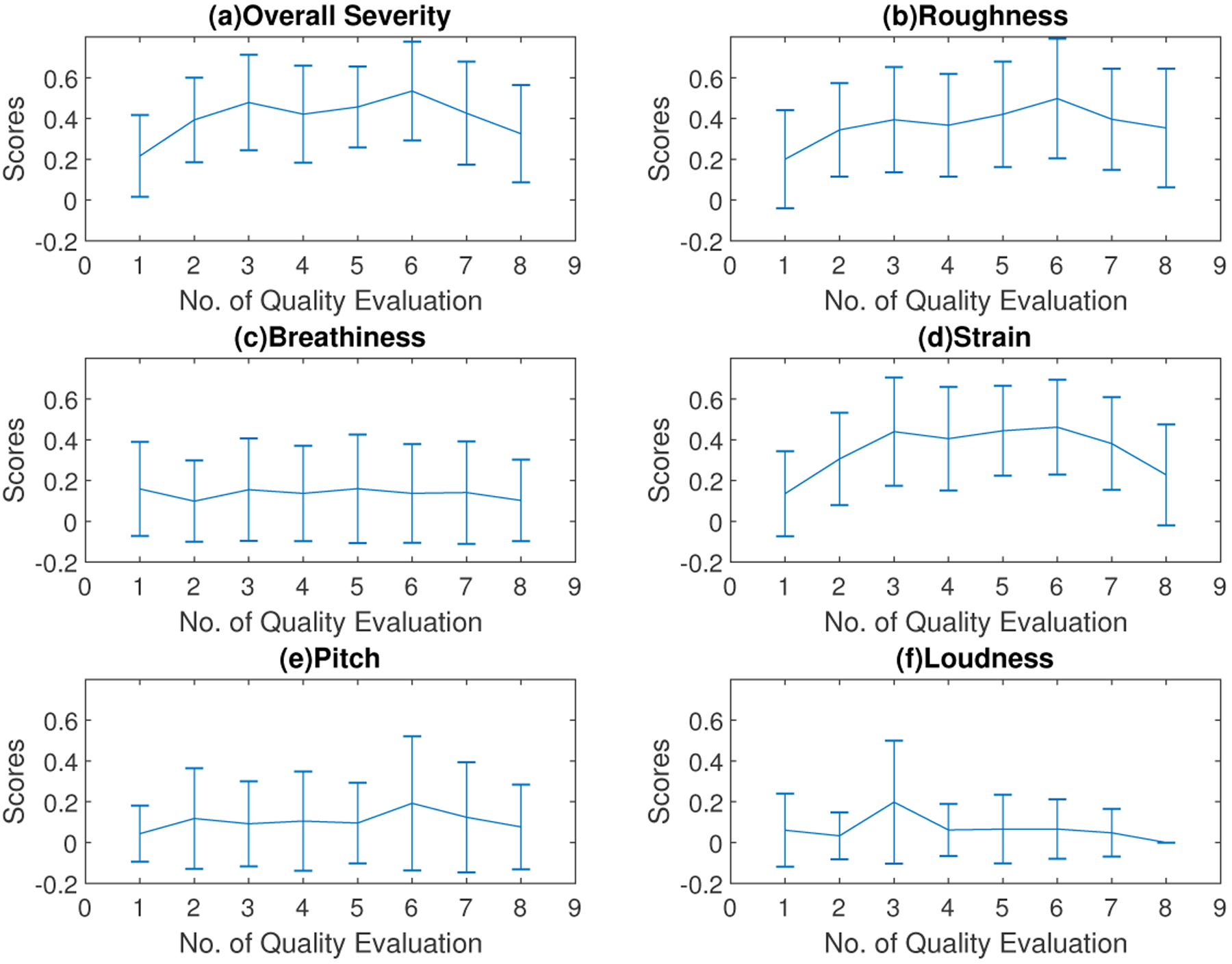
Cross-session variations mean and standard deviation values of CAPE-V ratings for all participants (n = 10).

The mean values and standard deviations of the normalized SAVRa and the original CAPE-V rating scores are shown in Figures 4 and 5, respectively. The Pearson correlation coefficients for SAVRa rating results (mean values) were 0.957 (EFFT vs. DISC), 0.834 (EFFT vs. IPSV), and 0.852 (DISC vs. IPSV), respectively. The average variation trajectories of these three dimensions are well correlated with each other. The EFFT and the DISC scores increased rapidly from QE1 to QE2, as shown in Figure 4. The IPSV score increased mildly at the beginning of the VLT sessions, which indicated that the soft phonation quality decreased slightly for S1. The average score increased for the EFFT, DISC, and IPSV from QE1 to QE2 were 0.52, 0.38, and 0.07, respectively. The effect of rest on self-reported voice quality ratings was notable for all dimensions. The mean and the standard deviation values of the three dimensions of the SAVRa ratings are shown in Table 3. The EFFT, the DISC, and the IPSV decreased by 50.0%, 59.3%, and 50.9%, respectively, from QE7 to QE8. In general, the high score ratings for all three dimensions occurred late in the sessions. The maximum (0.88) EFFT scores occurred at QE6, the maximum (0.88) DISC scores occurred at QE7, and the maximum (0.82) IPSV scores occurred at QE5. This indicated a cumulative effect, i.e., vocal fatigue increased over time during VLT sessions. In general, all three dimensions of the SAVRa ratings followed a similar trend across sessions.

The Pearson correlation coefficients between the overall severity trace and other dimensions in the CAPE-V ratings are shown in Table 4. This indicated that the overall severity, roughness, and strain variations were well correlated. The mean values of the overall severity, the roughness, and the strain scores followed a trend similar to that of the SAVRa results, as shown in Figure 5. They increased rapidly after S1 (QE1-QE3), remained constant for several sessions, and decreased over the remainder of the session. This trend was generally consistent with those of all three dimensions in SAVRa ratings, which showed a vocal ‘transition’ or ‘adjustment’ period in the first session and a vocal ‘recovery’ period during the final session. The maximum values of these three dimensions, shown in Figure 5, occurred at QE6. The mean and deviation values of the three dimensions of the SAVRa ratings are shown in Table 5. Other dimensions in the CAPE-V ratings followed different trends. The standard deviations of the CAPEV ratings across SLPs were larger than those of SAVRa ratings.

### 5.2. Vocal Fatigue Symptoms Recordings

**Figure 6.**
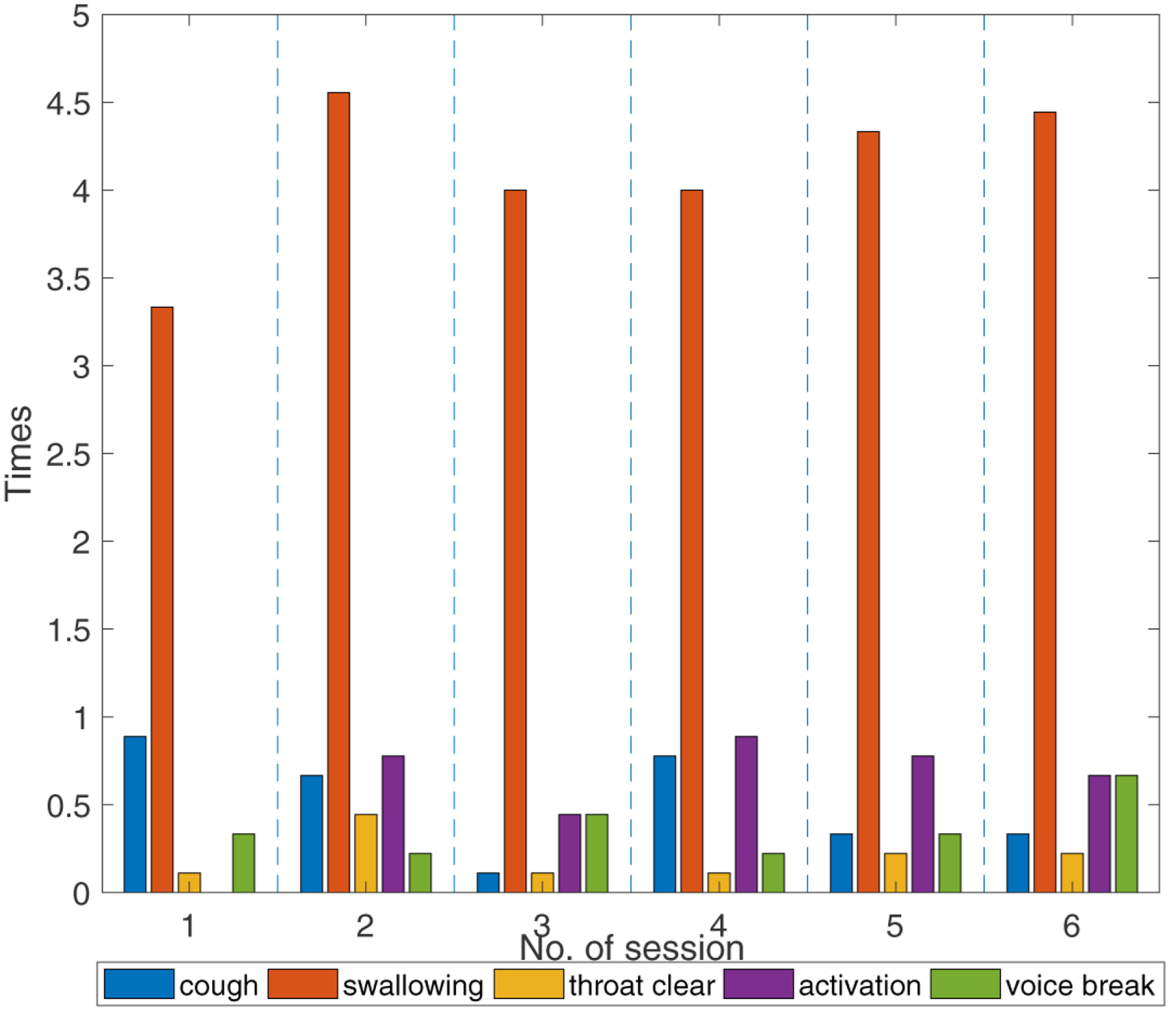
Mean counts of vocal fatigue symptom appearances for all participants during the VLTs in terms of session. The vertical blue dash lines separate different VLT sessions.

The participants’ vocal fatigue-indicative symptoms during the six VLT session are shown in Figure 6. The cross-participant discrepancy in the vocal symptoms is notable. For example, participant No. 4 was found to display much more (+47) symptoms than participant No. 8. One cough by participant No. 5 occurred for 4 VLT sessions, but no cough was observed for participants No. 3, No. 7, and No. 8 during any VLT sessions. The mean counts of vocal fatigue symptom appearances for all participants are shown in Figure 6. The average counts of swallowing increased by a factor of 1.8 from S1 to S2, then decreased to a relatively stable level (4–4.5 times). This trend is consistent with that of the overall severity, the roughness, and the strain in the CAPE-V ratings, and the EFFT in the SAVRa ratings. This finding validated the previous study results, which showed that re-hydration could relieve vocal fatigue, and allow voice to be sustained over longer time periods [44,45]. No similar trends were observed for any other symptoms, nor any dimensions in the CAPE-V and the SAVRa.

### 5.3. Acoustic Feature Analysis

**Figure 7.**
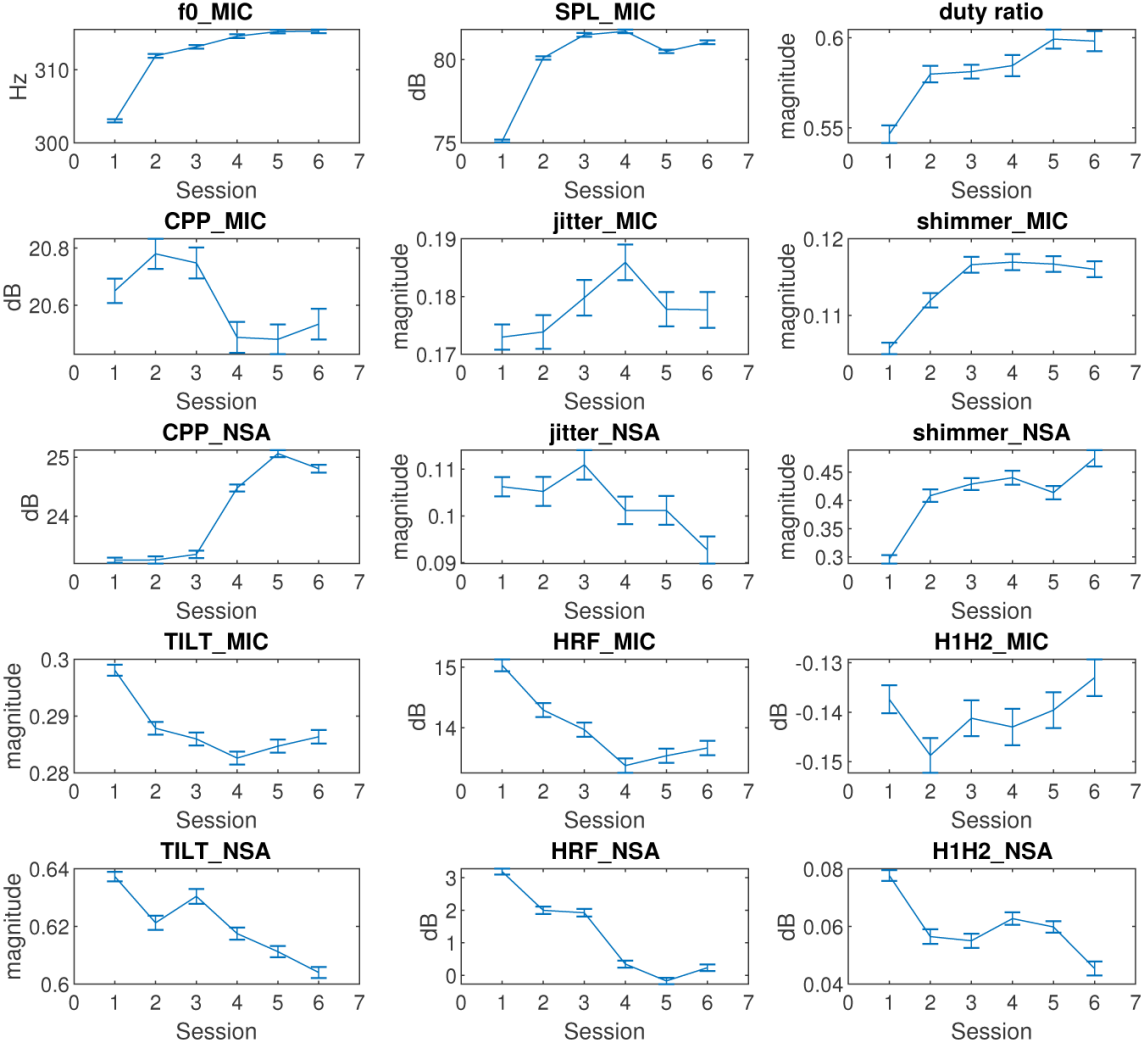
Cross-session variations of means and confidential levels in terms of feature for all participants.

The cross-session variations of the mean and confidence level (95%) of the features for all participants are shown in Figure 7. The NSA data of participant No.1 was unavailable due to sensor failure. The *f*0, SPL, and duty ratio in Figure 7 follow a similar trend. They increase rapidly at the beginning, reach a certain saturation level over the remaining sessions. *f*0, SPL, and duty ratio indicated a vocal adjustment over session S1. The mean value variations of these three features from S1 to S2 were at least 100% larger than those of any other following VLT session. The *f*0 had the minimal mean value variation (< 2 Hz) in the last three sessions. The SPL mean value decreased by 2 dB after S4 and increased by 1 dB after S5. The duty ratio mean value increased by 2% from one plateau (S2–S4) to another (S5–S6). The trends in *f*0, SPL and duty ratio were largely consistent with the trends in CAPE-V and SAVRa ratings.

A considerable decrease in CPP was found to be related to the presence of dysphonia by Heman-Ackah [16]. The CPP_MIC in Figure 7 showed a slight increase (0.15 dB) from S1 to S2, and a decrease (0.3 dB) from S3 to S4. The fluctuation of the CPP_MIC from S1 to S6 was less than 2%, which means that the variation of CPP_MIC was not significant. The CPP_NSA showed a sharp increase (2 dB) in the late session after sustaining a relatively low level for the first three sessions. The jitter_MIC increased rapidly from S1 to S4 by 10% but decreased by 5% afterwards, which was not well correlated with the subjective rating results for the VLT sessions. The jitter_NSA showed a trend similar to jitter_MIC, but with a larger decrease after S3. The mean values of the shimmer_MIC and shimmer_NSA were well correlated (*r* = 0.91, *p* = 0.987). Their progressions were similar to that of the *f*0. This indicates that the loudness perturbation increased rapidly in the early sessions, and saturated at a high level over the last sessions.

A decrease in spectral tilt slope was found to correlate with stressed phonation by Sluijter and Heuven [46]. A decrease in spectral tilt slope indicates that higher frequencies are increased more than lower frequencies. In Figure 7, the TILT_MIC slopes and the TILT_NSA slopes decreased from S1 to S6 in general. Thus, the high frequencies of both the microphone and NSA spectra increased throughout VLT sessions. Childers and Lee found that vocal fry had a higher HRF value than modal and breathy voice [47]. The HRF_MIC and the HRF_NSA obeyed similar trends than TILT_MIC and TILT_NSA. The HRF_MIC was well correlated (*r* = 0.94, *p* = 0.98) with HRF_NSA. These two features showed identical trends. The HRF mean values decreased from S1 and converged to S6. The H1H2_NSA decreased by 50% from S1 to S6, and remained constant from S2 to S5. A comprehensive analysis of the Pearson correlation between each pair of features is shown in Table 6. There are two groups of well correlated (> 90%) features. The first group includes *f*0, SPL_MIC, duty ratio, shimmer_MIC, and shimmer_NSA. The first group shows a rapid increase in early session followed by saturation. The second group includes TILT_MIC, TILT_NSA, HRF_MIC, and HRF_NSA and shows a general decrease from S1 to S6.

## 6. Discussion and Conclusion

The primary finding in the SAVRa results was that the ratings show an arch-shaped variation trajectory from QE1 to QE8. This trend was also observed in the CAPE-V rating results. In the SAVRa ratings, the rapid increase of the vocal effort, the vocal discomfort level, and the severity of the soft phonation quality indicated a voice adjustment period. Over this period, the participants’ vocal folds were abruptly exposed to a heavy vocal loading, and thus the subjects’ feelings of vocal fatigue were strong over this period. After this period, the degrees of the participants’ perceived vocal fatigue remained constant at a high level, or increased moderately from QE2 to QE7. The vocal loading intensity for each VLT session was identical. The participants gradually adapted to the vocal loading intensity, thus the increasing rate slowed down after this period. After the S1, the accumulation effect of the vocal loading led to a slow and persistent increase of the participants’ perceived vocal fatigue in the late sessions. This indicates that, given constant vocal stimuli for different sessions, the participants’ perceived vocal fatigue increased over time (or vocal distance dose). The notable decrease of the SAVRa scores from QE7 to QE8 reflects the effect of vocal rest on participants’ vocal fatigue feelings. This indicates that the participants felt much better about their vocal functionalities after the 15-minute rest session, but the rest was not sufficient to completely recover to the original status at QE1. A longer rest session was thus presumed to enhance the vocal recovery process. The findings in the CAPE-V rating results show that the overall severity, roughness, and strain ratings have the same arch-shaped variation trend with the SAVRa results. This indicates that vocal fatigue degraded the participants’ voice performance by increasing roughness and strain.

The data for the fatigue-indicative symptoms obeyed different trends than the SAVRa results. Previous studies showed that these symptoms were frequently observed on vocally fatigued participants. However, a quantitative relationship between the number of occurrences of these symptoms and the degree of vocal fatigue could not be established. Individual discrepancies in the counts of these symptoms showed varied sensitivities to vocal fatigue. Some participants coughed frequently during the VLT, while others did not cough at all. Swallowing was much more frequently observed than other symptoms for all participants. This finding further validated the results of previous studies on the effect of hydration on vocal fatigue [44,48], i.e., superficial vocal fold hydration (swallowing or drinking) could help relieve vocal dysfunction and improve vocal efficiency.

Fifteen voice metrics (features) were studied to track voice quality variation across VLT sessions. The statistical analysis results in Figure 7 did not include the rest session. The *f*0, SPL_MIC, duty ratio, shimmer_MIC, and shimmer_NSA rapidly increased in early sessions, and remained constant afterwards. The variation of *f*0 in Figure 7 was consistent with the results of previous studies, which found that participants’ *f*0 increased with vocal fatigue [49,50]. The shimmer_MIC and shimmer_NSA showed an increase after the VLT session. This finding contradicts Laukkanen’s one-day vocal fatigue study results [51], but it is consistent with Gelfer’s 60-minute vocal fatigue study results [49]. This might be caused by the different phonation durations.

One limitation of the present study was that the voice type was not considered as a parameter in the voice use quantification. For example, breathy voice obviously had a different vocal loading intensity from pressed voice when the *f*0, SPL, duty ratio, and duration were identical for these two voice types. The impact stress between vocal folds for these two voice types were different. The feelings of vocal fatigue that the breathy and pressed voice brought to participants were therefore different. An improved method of calculating *D*_*d*_ that considers voice quality is therefore needed to make the voice use quantification more accurate. Another limitation was that calculation of the *D*_*d*_ from the NSA may be not very accurate because of the rather large uncertainty in the SPL (± 5 dB) estimates from the NSA data [40]. The accuracy of calculating *D*_*d*_ would thus be influenced. This issue limits the use of the dose-based voice use quantification method in long-term voice monitoring. The monitoring of neck surface acceleration is preferable to minimize the influence of extraneous noise and potentially reduce discomfort in occupational settings. A method for directly deriving the magnitude of the vocal fold vibration from the skin acceleration level is therefore needed. The last limitation is that normal swallowing on a daily basis was not measured, and thus the fatigue-indicative symptom analysis lacked a baseline to refer to. This baseline could be established in future work.

## Figures and Tables

**Table 1.** Personal information about the participants.

ID	Age	Occupation
1	32	SLP
2	22	psychology student
3	38	psychology student
4	25	engineering student
5	23	SLP student
6	22	nutrition student
7	24	SLP student
8	26	engineering student
9	31	arts student
10	26	psychology student

**Table 2.** List of features extracted from the microphone and NSA signals.

Symbols	Explanation	Source
*f*0	fundamental frequency	microphone
SPL	sound pressure level	microphone
duty ratio	voicing percentage in recording time	microphone
CPP_MIC	cepstral peak prominence	microphone
jitter_MIC	pitch perturbation	microphone
shimmer_MIC	loudness perturbation	microphone
CPP_NSA	cepstral peak prominence	NSA
jitter_NSA	pitch perturbation	NSA
shimmer_NSA	loudness perturbation	NSA
TILT_MIC	spectral tilt	microphone
HRF_MIC	harmonic richness factor	microphone
H1H2_MIC	different between H1 and H2 magnitudes	microphone
TILT_NSA	spectral tilt	NSA
HRF_NSA	harmonic richness factor	NSA
H1H2_NSA	different between H1 and H2 magnitudes	NSA

**Table 3.** SAVRa mean and deviation values in terms of VLT session. The display format was *x*(*y*), where *x* was the mean and *y* was the standard deviation. QEi(i=1…8): quality evaluation. EFFT: speaking effort level. DISC: laryngeal discomfort level. IPSV: inability to produce soft voice.

	EFFT	DISC	IPSV
QE1	0.08(0.25)	0.02(0.07)	0.23(0.39)
QE2	0.61(0.38)	0.40(0.27)	0.30(0.28)
QE3	0.54(0.20)	0.52(0.27)	0.46(0.28)
QE4	0.66(0.27)	0.48(0.22)	0.71(0.31)
QE5	0.80(0.14)	0.76(0.13)	0.82(0.19)
QE6	0.88(0.25)	0.83(0.14)	0.67(0.30)
QE7	0.84(0.26)	0.88(0.26)	0.72(0.36)
QE8	0.42(0.38)	0.36(0.32)	0.35(0.45)

**Table 4.** Pearson correlation coefficients between the overall severity and other dimensions.

With Overall Severity	*r*	*p*
Roughness	0.948	0.00
Breathiness	0.103	0.81
Strain	0.969	0.00
Pitch	0.810	0.01
Loudness	0.402	0.32

**Table 5.** CAPEV mean and deviation values in terms of VLT session. The display format was *x*(*y*), where *x* was the mean and *y* was the standard deviation. OS: overall severity, RG: Roughness, BT: Breathiness, ST: Strain, PT: Pitch, LD: Loudness.

	OS	RG	BT	ST	PT	LD
QE1	0.22(0.20)	0.20(0.24)	0.16(0.23)	0.14(0.21)	0.04(0.14)	0.06(0.18)
QE2	0.39(0.21)	0.34(0.23)	0.10(0.20)	0.31(0.23)	0.12(0.25)	0.03(0.12)
QE3	0.48(0.23)	0.39(0.26)	0.15(0.25)	0.44(0.27)	0.09(0.21)	0.20(0.30)
QE4	0.42(0.24)	0.37(0.25)	0.14(0.23)	0.41(0.25)	0.11(0.24)	0.06(0.13)
QE5	0.46(0.20)	0.42(0.26)	0.16(0.27)	0.44(0.22)	0.10(0.20)	0.07(0.17)
QE6	0.53(0.24)	0.50(0.29)	0.14(0.24)	0.46(0.23)	0.19(0.33)	0.07(0.15)

**Table 6.** Pearson correlation coefficients for each pair of features. The fi(i = 1, …, 15) represents *f*0, SPL, duty ratio, CPP_MIC, jitter_MIC, shimmer_MIC, CPP_NSA, jitter_NSA, shimmer_NSA, TILT_MIC, HRF_MIC, H1H2_MIC, TILT_NSA, HR_NSA, and H1H2_NSA, respectively.

	f1	f2	f3	f4	f5	f6	f7	f8	f9	f10	f11	f12	f13	f14	f15
f1	1	*	*	*	*	*	*	*	*	*	*	*	*	*	*
f2	0.95	1	*	*	*	*	*	*	*	*	*	*	*	*	*
f3	0.97	0.86	1	*	*	*	*	*	*	*	*	*	*	*	*
f4	−0.39	−0.20	−0.45	1	*	*	*	*	*	*	*	*	*	*	*
f5	0.61	0.69	0.44	−0.51	1	*	*	*	*	*	*	*	*	*	*
f6	0.96	0.96	0.90	−0.40	0.74	1	*	*	*	*	*	*	*	*	*
f7	0.68	0.46	0.76	−0.91	0.46	0.63	1	*	*	*	*	*	*	*	*
f8	−0.44	−0.24	−0.56	0.70	−0.15	−0.30	−0.78	1	*	*	*	*	*	*	*
f9	0.95	0.94	0.90	−0.28	0.59	0.91	0.57	−0.51	1	*	*	*	*	*	*
f10	−0.96	−0.97	−0.87	0.37	−0.75	−0.96	−0.59	0.29	−0.89	1	*	*	*	*	*
f11	−0.95	−0.89	−0.89	0.62	−0.79	−0.95	−0.79	0.49	−0.87	0.95	1	*	*	*	*
f12	−0.06	−0.20	0.08	−0.50	−0.04	−0.01	0.45	−0.54	0.03	0.22	−0.03	1	*	*	*
f13	−0.80	−0.63	−0.89	0.62	−0.30	−0.67	−0.86	0.87	−0.79	0.67	0.77	−0.26	1	*	*
f14	−0.88	−0.72	−0.91	0.77	−0.59	−0.83	−0.94	0.69	−0.77	0.82	0.94	−0.21	0.90	1	*
f15	−0.82	−0.80	−0.83	0.03	−0.23	−0.73	−0.40	0.47	−0.92	0.68	0.63	−0.10	0.73	0.58	1

## Abbreviations

CAPE-V: Consensus Auditory-Perceptual Evaluation of Voice
SAVRa: Self-Administrated Voice Rating
GRABAS: Gradem Roughness, Breathiness, Asthenia, Strain
NSA: Neck Surface Accelerometer
SLP: Speech Language Pathologist
MFDR: Maximum Flow Declination Rate
MFCC: Mel-Frequency Spectral Coefficients
VLT: Vocal Loading Task
EGG: Electroglottograph
SPL: Sound Pressure Level
CIRMMT: Centre for Interdisciplinary Research in Music Media and Technology
QE: Quality Evaluation
IPSV: Inability to Produce Soft Voice
CPP: Cepstral Peak Prominence
HRF: Harmonic Richness Factor
